# Disparities in robot utilization in colorectal surgery: the widening gap

**DOI:** 10.1007/s00464-025-12459-w

**Published:** 2026-07-08

**Authors:** Andrew E. Donaldson, Sarah B. Jochum, Jimmie Knight, Joshua M. Underhill, Chassidy Grimes, Laura A. DeCesare, Brendan O’Donnell, Ethan M. Ritz, Henry R. Govekar, Anuradha R. Bhama, Erin King-Mullins, Dana M. Hayden

**Affiliations:** 1 Department of Surgery, Rush University Medical Center, 1620 W. Harrison St, Chicago, IL 60612 USA; 2 Rush Bioinformatics and Biostatistics Core, Chicago, IL USA; 3 Diversity in Colon and Rectal Surgery, American Society of Colon and Rectal Surgeons, Chicago, IL USA

**Keywords:** Racial disparities, Robotic surgery, Minimally invasive surgery, NSQIP, Colectomy, Proctectomy

## Abstract

**Background:**

Robot-assisted surgery has expanded in adoption and indication among multiple surgical specialties, including colorectal surgery. It is unclear if access to robotic surgery is increasing equally among all patient populations.

**Objective:**

(1) Identify the factors that may influence type of surgical approach and (2) evaluate disparities in access and subsequent outcomes of colectomy and proctectomy from years 2013–2020 using a national database.

**Study design:**

We performed a retrospective study using the National Surgical Quality Improvement Program to identify adult patients who had undergone colectomy and/or proctectomy from years 2013–2020. We performed multivariate analysis to identify factors that may influence the likelihood of receiving robotic surgery.

**Settings:**

Tertiary care medical center.

**Patients:**

18 years and older, received colectomy and/or proctectomy from 2013–2020.

**Main outcome measures:**

Frequency of robotic colorectal surgery, postoperative complications, and mortality.

**Results:**

A total of 125,776 patients met inclusion criteria. Mean age was 61.4 years; 12.8% (16,086) of patients were non-White. In multivariate analysis, patients who were female, non-White, underweight or had inflammatory bowel disease were less likely to undergo robot-assisted surgery (p < 0.05). Although the total number of robotic cases increased year-to-year, the gap between White and non-White patients widened. In 2014, a White patient was 1.51 times more likely to have robot-assisted surgery compared to a non-White patient; odds increased to 1.96 in 2020 (p < 0.001). Patients who underwent robot-assisted surgery were less likely to have a postoperative complication (OR 0.86, CI 0.82–0.9) or serious adverse event (OR 0.9, CI 0.84–0.97), and had lower odds of 30-day mortality (OR 0.72, CI 0.57–0.91).

**Limitations:**

Retrospective, limited coding in national databases, and database variables.

**Conclusions:**

Despite increased awareness of racial and ethnic disparities in surgical care, non-White patients’ decreased access to robot-assisted surgery is troublesome. Interventions must focus on closing this gap if we are to provide equitable healthcare.

Since its initial design and subsequent FDA approval, robot-assisted surgery (RAS) developed footholds in numerous surgical specialties and continues to grow in its application. From its beginnings, robotics has demonstrated value in the low pelvic dissections required for prostatectomies, three-dimensional optics, improved dexterity and tremor elimination, which have proven beneficial in colorectal surgery as well [1–4]. Although adoption of robotics into colorectal surgery was initially behind the pace of other specialties, its use on a national scale has steadily increased from the early 2000s to present [5–11]. The short-term outcomes of RAS compared to laparoscopy for management of colorectal diseases have largely proven to be equivalent. In fact, some reports cite decreased rates of postoperative complications, lower conversion rates, shorter hospital length of stay (LOS) and equivalent oncologic outcomes in RAS compared to laparoscopy. [1, 2, 11, 12]

As would be expected with a technology as expensive as the robotic surgical platform, its increased utilization has largely been observed in academic teaching hospitals in metropolitan areas rather than community-based or rural hospitals [13–15]. This skewed distribution of RAS availability set the stage for further disparities, particularly with regards to patient race/ethnicity and socioeconomic status. Colorectal surgery as a specialty has seen racial disparities in both access to minimally invasive surgery (MIS) and postoperative outcomes for benign and malignant colorectal disease. It is well-documented in the literature that Black patients are offered MIS less often compared to their White counterparts as well as experience persistently higher rates of postoperative complications and mortality in some circumstances [16–21]. Despite widespread increased awareness of disparities in healthcare, these racial inequities seem to exist in access to robotic surgery as well, with recent studies demonstrating that RAS may be offered more frequently to White patients or those with private insurance. [10, 13, 22–24]

To our knowledge, a study of national trends in access to robotic colorectal surgery as compared to other approaches over time has yet to be conducted. One study examined trends and disparities in overall usage of several surgical procedures at the national level; for colorectal resection, the gap between Black and White patients fortunately decreased over time [16]. However, it remains unclear if the use of robotic surgery is increasing equally in all patient populations. The objective of this study is to (1) identify the factors that may influence type of surgical approach and (2) evaluate disparities in access and subsequent outcomes of colectomy and proctectomy from years 2013–2020 using a national database.

## Methods

### Data source and patient selection

The American College of Surgeons National Surgical Quality Improvement Program (ACS NSQIP) is a validated, risk-adjusted, nationwide database that contains data on surgical patients and their outcomes within 30 days of surgery. Using NSQIP, we identified patients 18 years and older who had undergone colectomy and/or proctectomy for any reason from years 2013–2020. We included patients who underwent resection via open, laparoscopic, or robot-assisted approach. Surgeries performed in an emergency setting and surgeries performed endoscopically or via a hybrid approach were excluded. We identified our patients of interest using the International Classification of Diseases (ICD) -9 and ICD-10 diagnosis codes and Current Procedural Terminology (CPT) codes, as described in Appendix 1. The study protocol was reviewed by the Rush University Medical Center Institutional Review Board, which was provided exemption status.

## Outcomes

Once our full cohort of patients was identified, we separated them into three groups based on surgical approach: open, laparoscopic or robotic. Demographic and clinical factors were compared between these groups to identify any potential predictors for robotic versus non-robotic (open or laparoscopic) surgery. Variables included demographic and clinical factors such as sex, age, race/ethnicity, comorbidities existing at the time of surgery, diagnosis group (colorectal malignancy, inflammatory bowel disease [IBD], or benign), and type of surgery (colectomy or proctectomy). NSQIP does not include data on indication for surgery, so diagnosis group was used as a surrogate for this variable. The primary outcome for this study was frequency of robotic colorectal surgery. Secondary outcomes included postoperative complications and mortality.

An important aim in our investigation was the identification of any trends in robotic surgery utilization over time. As such, we performed our statistical analyses for both the entire study duration (2013–2020) and on an annual basis. In particular, we compared annual frequencies of robotic versus non-robotic (open or laparoscopic) approach and robotic versus laparoscopic approach in White versus non-White patients. Then, we performed a sub-analysis in which we separated non-White into the constituent individual races (American Indian or Alaska Native, Asian, Black or African American, Native Hawaiian or Pacific Islander, and Unknown/Not Reported) and again compared those groups to White patients.

## Statistical analysis

Categorical data was presented as total number and percentage frequency while continuous data were presented as mean values ± standard deviation. Any missing data were excluded from analysis. Categorical variables were compared using χ^2^ or Fischer’s exact test and continuous data were compared using the Kruskal–Wallis test. We performed univariate and multivariate analysis to identify the association between any variables with open, laparoscopic or robotic approaches. For all multivariate analysis, covariates were selected using a backward elimination procedure and can be found in Appendix 2. We reported odds ratios (OR) with the corresponding 95% Wald confidence interval (CI). For all analyses, we defined statistical significance as p < 0.05. The Bonferroni correction was applied when making multiple simultaneous comparisons. All analyses were performed with SAS version 9.4 (Table 1).

**Table 1:**
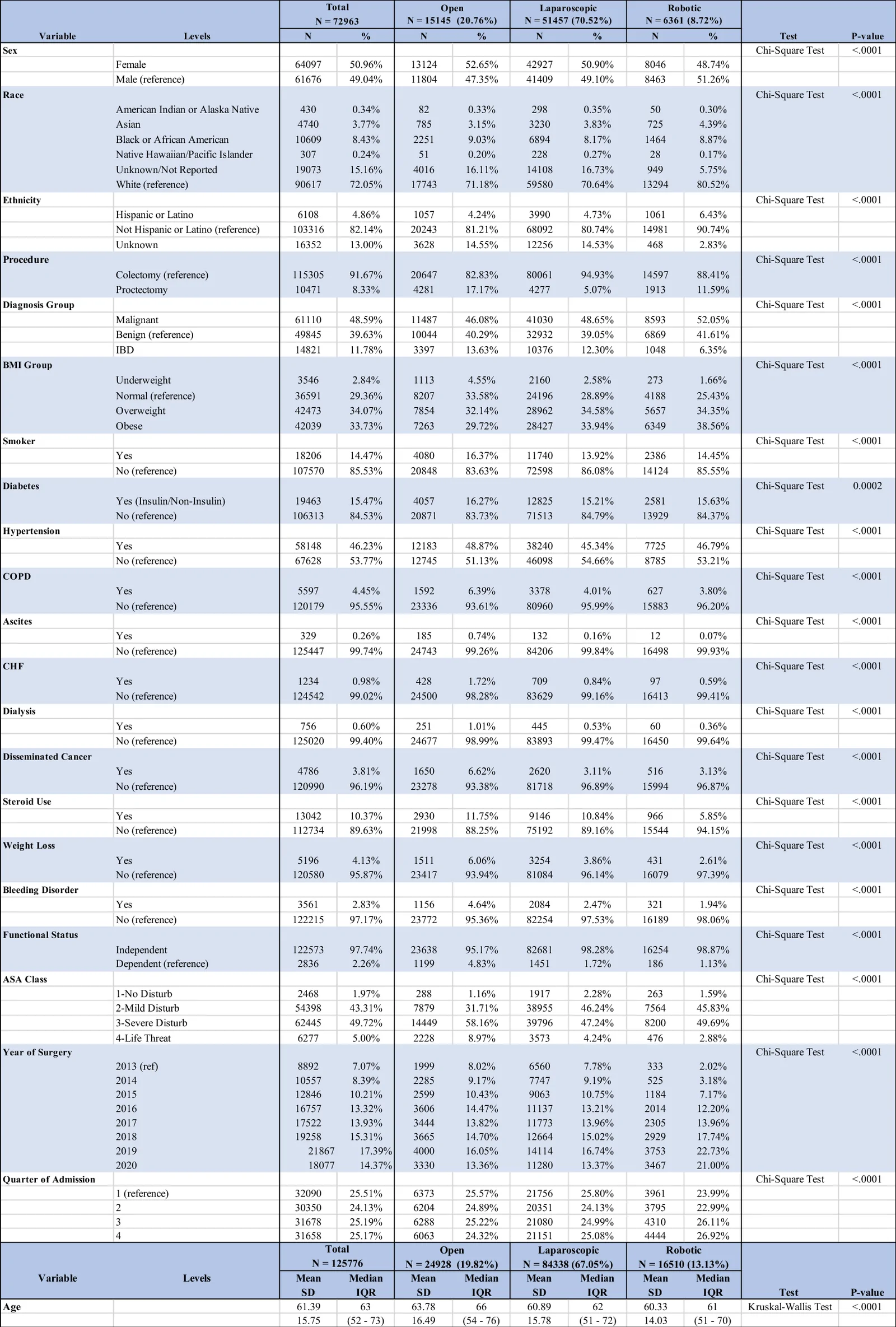
NSQIP patient characteristics

## Results

### Patient characteristics

Demographic and clinical characteristics of the patient population are listed in Table 2 and 3. A total of 125,776 patients met inclusion criteria. The mean age was 61.4 years and 51% (n = 64,097) of patients were female. The majority of patients were reported as White (72.1%, n = 90,617), while non-White patients represented 12.8% (16,086) of the population. The remainder of the population had race unknown or was not reported (15.2%, n = 19,073). Black patients represented 8.4% (n = 10,609) of the entire population and 66% of the non-White patients.

**Table 2:**
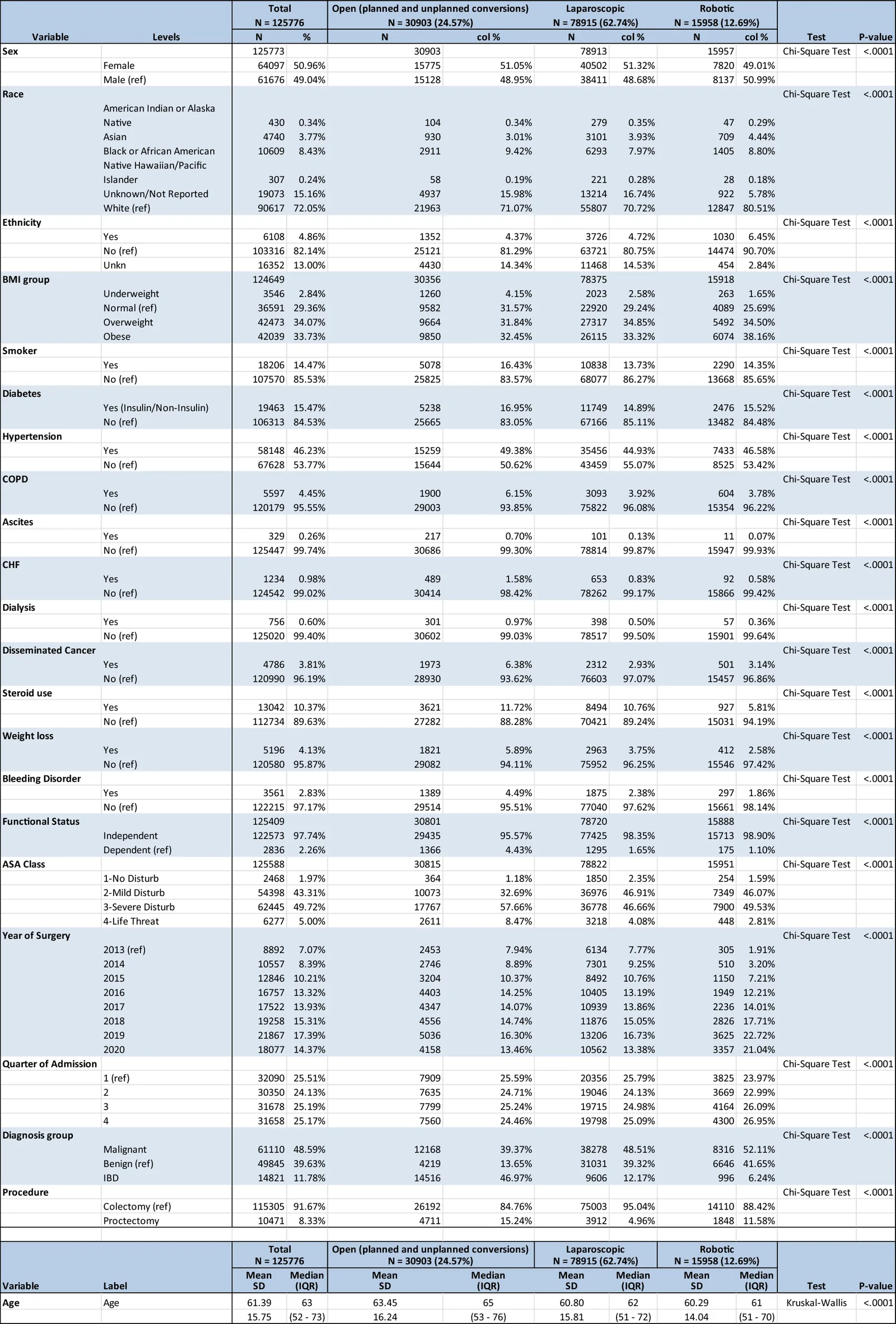
NSQIP multivariate analysis: odds of having undergone robotic vs non-robotic (open or laparoscopic) surgery in Whites versus non-Whites

**Table 3:**
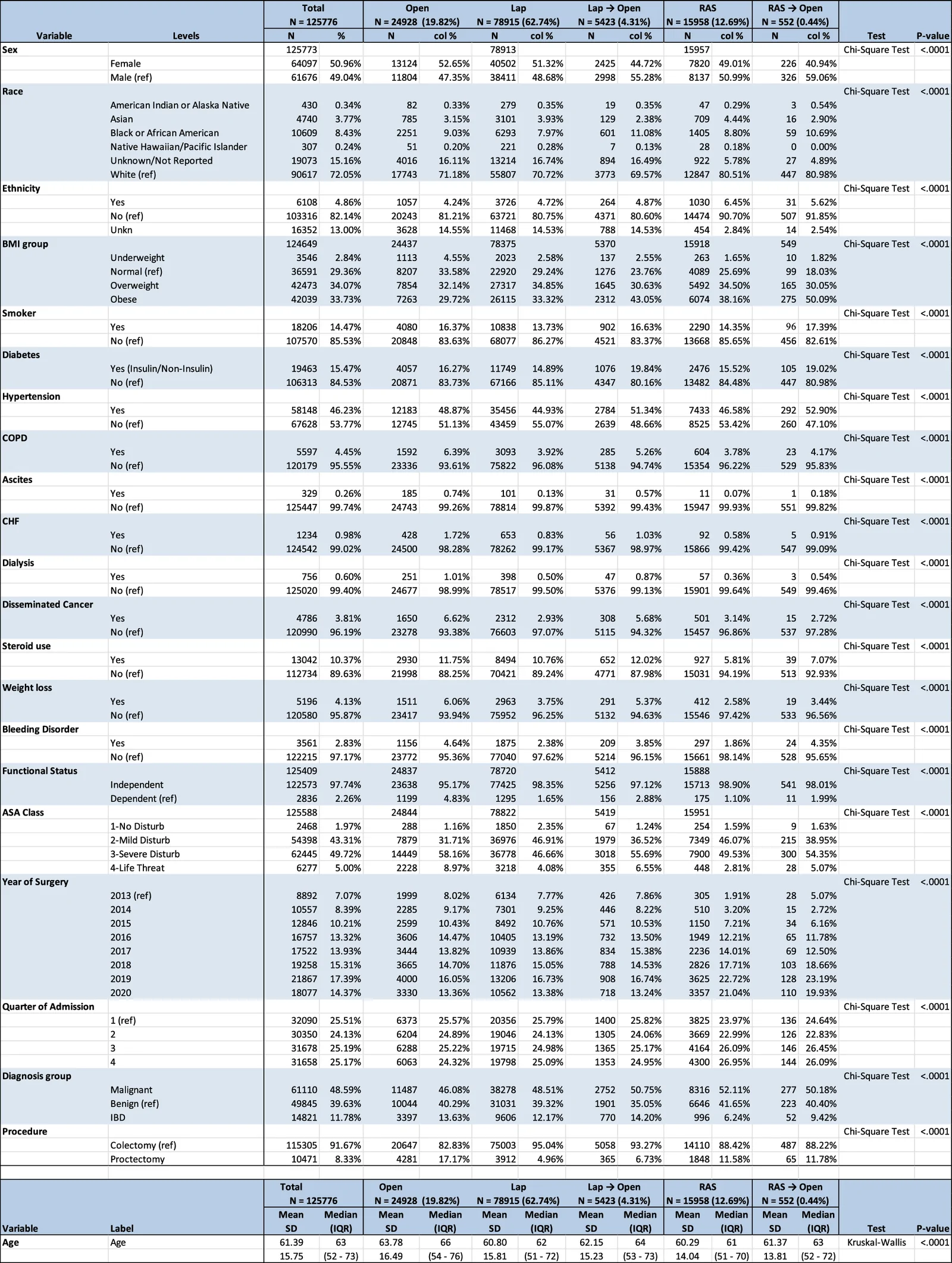
Patient demographics separated into unplanned conversions to open (lap and robotic)

Almost all (91.7%, n = 115,305) resections were classified as colectomies; 8.3% (n = 10,471) were proctectomies. For approach, 19.8% (n = 24,928) of surgeries were performed open, 67.1% (n = 84,338) were performed laparoscopically, and 13.1% (n = 16,510) were robot-assisted. Malignancy was the most common diagnosis (48.6%, n = 61,110), followed by benign disease (39.6%, n = 49,845) and IBD (11.8%, n = 14,821). Of note the database accounted for robotic and laparoscopic procedures with planned and unplanned conversion to open without changes in statistical significance (Table 2 and 3). The complete list of comorbidities existing at the time of surgery are listed in Table 2 and 3.

## Comparing robotic versus non-robotic surgery

Using multivariate analysis, we identified several patient factors that were significantly associated with either higher or lower odds of undergoing RAS compared to non-robotic surgery (Table 4). Females (OR 0.91, CI 0.88–0.95) and non-White patients (OR 0.82, CI 0.78–0.86) had decreased odds of undergoing RAS compared to males and White patients, respectively. Ethnicity did not significantly influence a patient’s odds. Patients who underwent proctectomy were much more likely (OR 1.65, CI 1.56–1.74) to have their resection performed robotically than colectomy patients. Compared to benign colorectal disease, those with a colorectal malignancy were more likely to undergo RAS (OR 1.16, CI 1.12–1.21) while those with IBD were less (OR 0.38, CI 0.35–0.41). Underweight patients were less likely to have RAS (OR 0.72, CI 0.63–0.82) while overweight and obese patients more often had robotic surgery (OR 1.11, CI 1.06–1.16 and OR 1.2, CI 1.15–1.26, respectively). Odds significantly decreased for almost every individual comorbidity, including current smoker, presence of ascites, history of CHF, disseminated cancer, steroid use, weight loss, and bleeding disorder (see Table 4 for ORs and p values). Relative to ASA class 1 patients, classes 2 and 3 were more likely to undergo robotic surgery (OR 1.21, CI 1.06–1.39 and OR 1.16, CI 1.01–1.33, respectively), while class 4 were less likely (OR 0.83, CI 0.7–0.98). Across the entire cohort, odds of undergoing RAS increased on an annual basis (OR 1.26, CI 1.25–1.27).

**Table 4:**
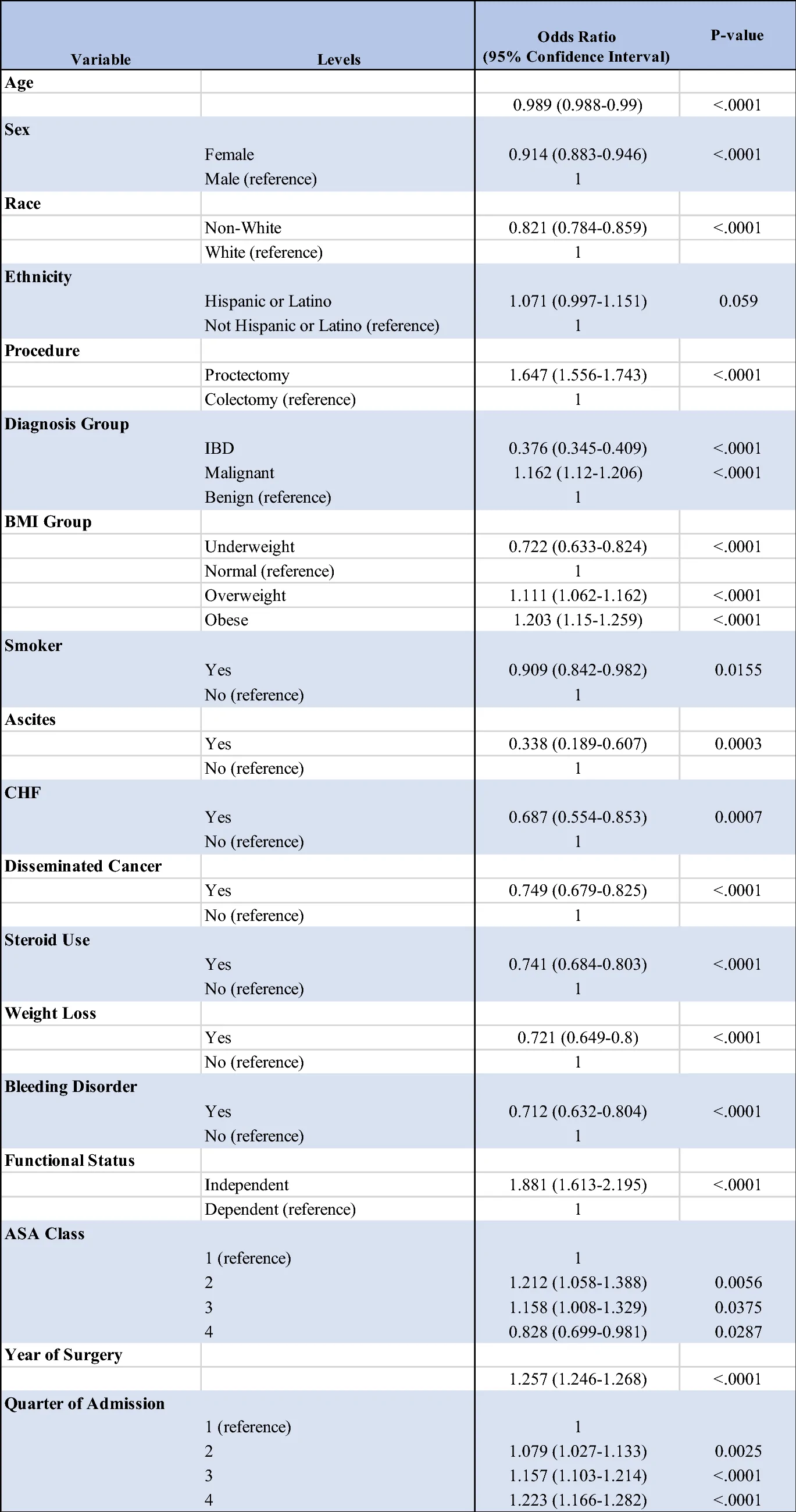
NSQIP multivariate analysis: odds of having undergone robotic vs non-robotic (open or laparoscopic) surgery in Whites versus non-Whites

## Annual trends in access to robotic surgery

**Fig. 1:**
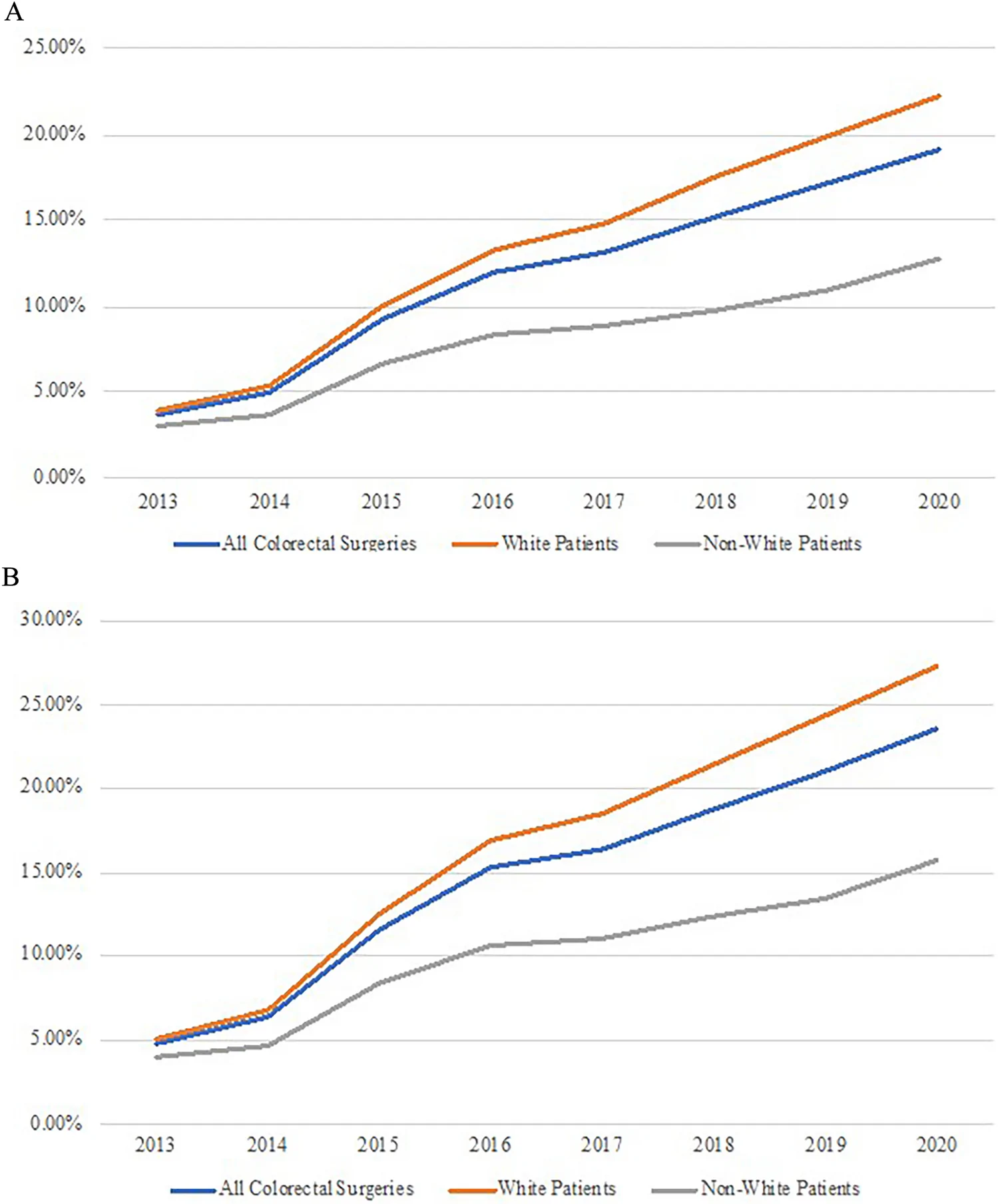
**A** compares the percentage of all colorectal surgeries that were done robotically between White and non-White patients, clearly showing a widening gap as time goes by. **B** examines only colorectal surgeries that were done in a minimally-invasive manner; the percentage of surgeries done robotically increases disproportionately for White patients compared to non-White patients as time goes on

We separated the data by year of surgery and again compared White and non-White patients to identify any potential trends in racial disparities. Comparing robotic versus non-robotic surgery again demonstrated that non-White patients were significantly less likely to undergo robotic surgery (Table 5). In 2014, White patients were 1.51 times more likely to have robotic surgery (CI 1.19–1.91). These odds steadily increased to their maximum in 2019: 2.03 (CI 1.86–2.21). The odds in 2020 remained very similar, at 1.96. These data are represented graphically in Fig. 1A, which depicts the overall increase in percentage of surgeries performed robotically and the increasing gap between White and non-White patients.

**Table 5:**
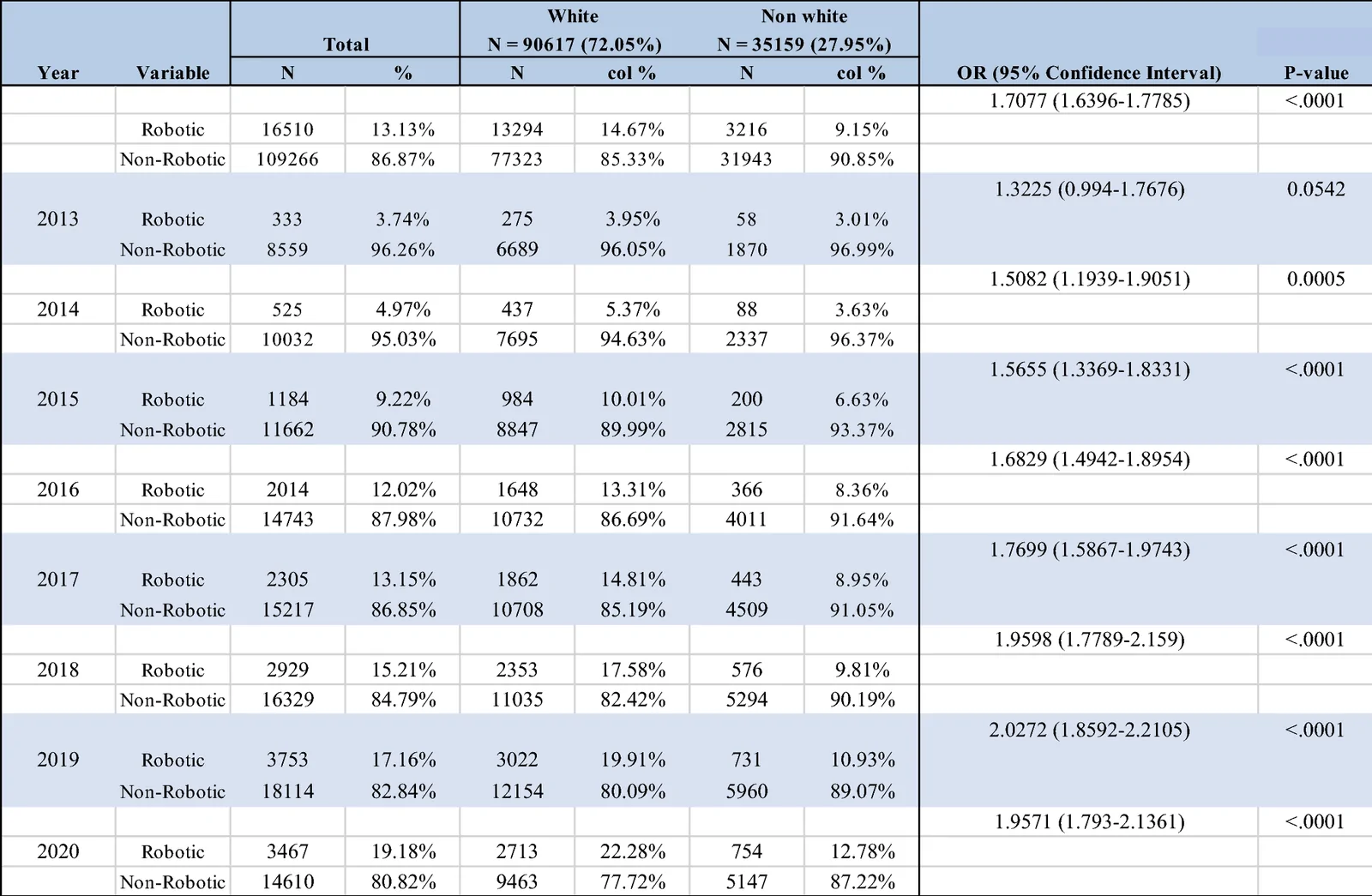
NSQIP multivariate analysis: Annual odds of undergoing robotic surgery in White vs non-White patients

Sub-analysis of MIS surgeries (robotic or laparoscopic only) yielded very similar results. In 2014, White patients were 1.48 times more likely to have robotic surgery when compared to laparoscopic (CI 1.17–1.87) and 2.02 times more likely in 2020 (CI 1.85–2.21) (Table 6). This trend is represented graphically in Fig. 1.

**Table 6:**
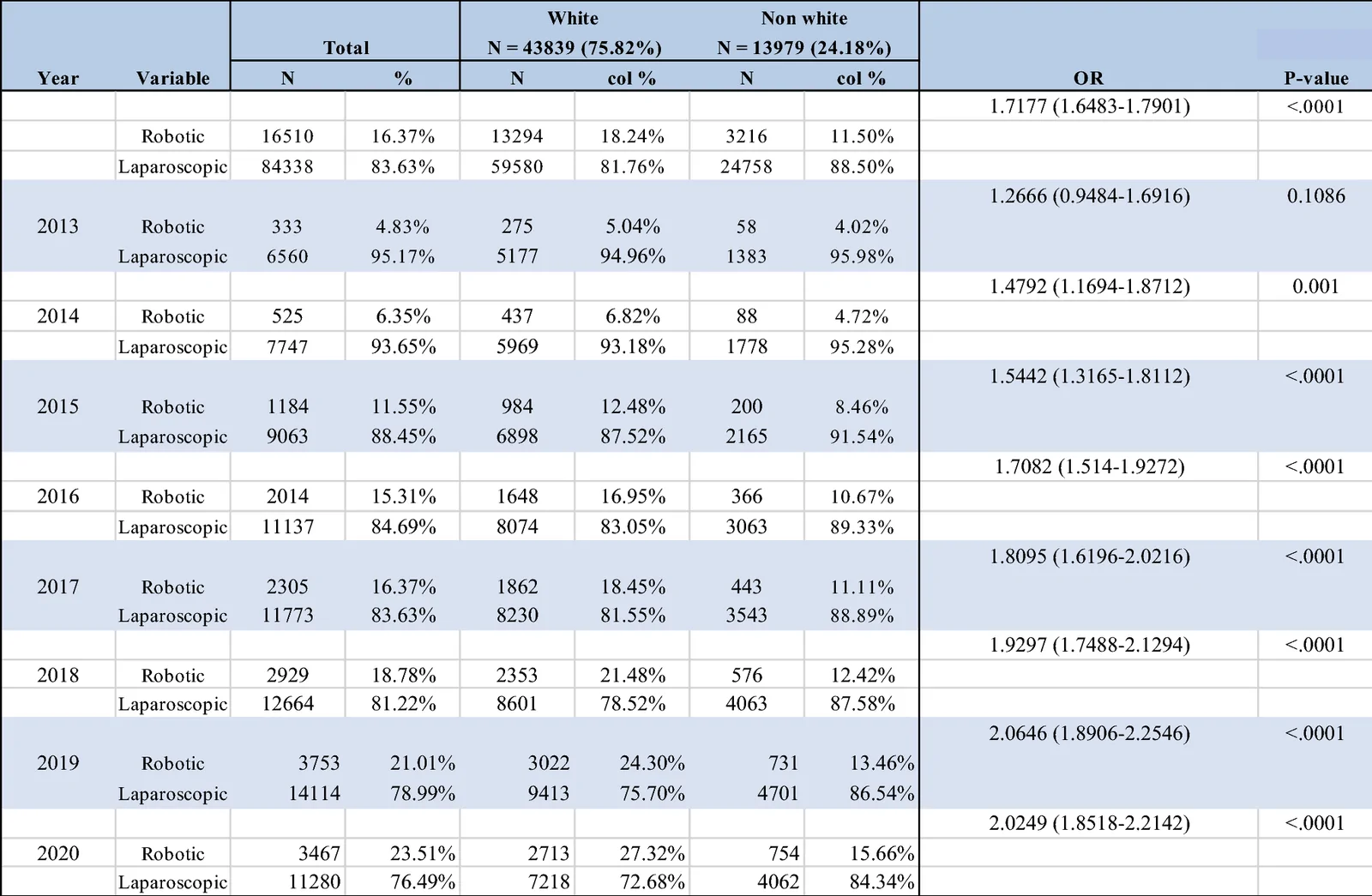
NSQIP multivariate analysis: annual odds of undergoing robotic vs laparoscopic surgery in White vs non-White patients

## Sub-analysis by patient race

In the final sub-analysis, the “non-White” group was categorized separately into separate racial groups, including American Indian or Alaska Native, Asian, Black or African American, Native Hawaiian or Pacific Islander, and Unknown/Not Reported. We compared access to robotic surgery in each of these individual racial groups to White patients, both overall and annually. Multivariate analysis demonstrated that all non-White racial groups had significantly lower odds of undergoing robotic surgery over the duration of the study period, with ORs ranging from 0.51 to 0.93 (Table 7). Analysis also demonstrated that White patients had significantly higher odds of undergoing MIS surgery overall for all years of the study period (OR 1.05, 1.02–1.09, p < 0.05) (Table 8). Furthermore, non-White patients had significantly higher odds of undergoing an unplanned conversion to open (OR:1.06, 0.995–1.12, p < 0.05) (Table 9). When evaluating odds of RAS by racial group and year, no definitive trends could be identified for either robotic versus non-robotic or robotic versus laparoscopic surgery (Appendices 3 and 4).

**Table 7:**
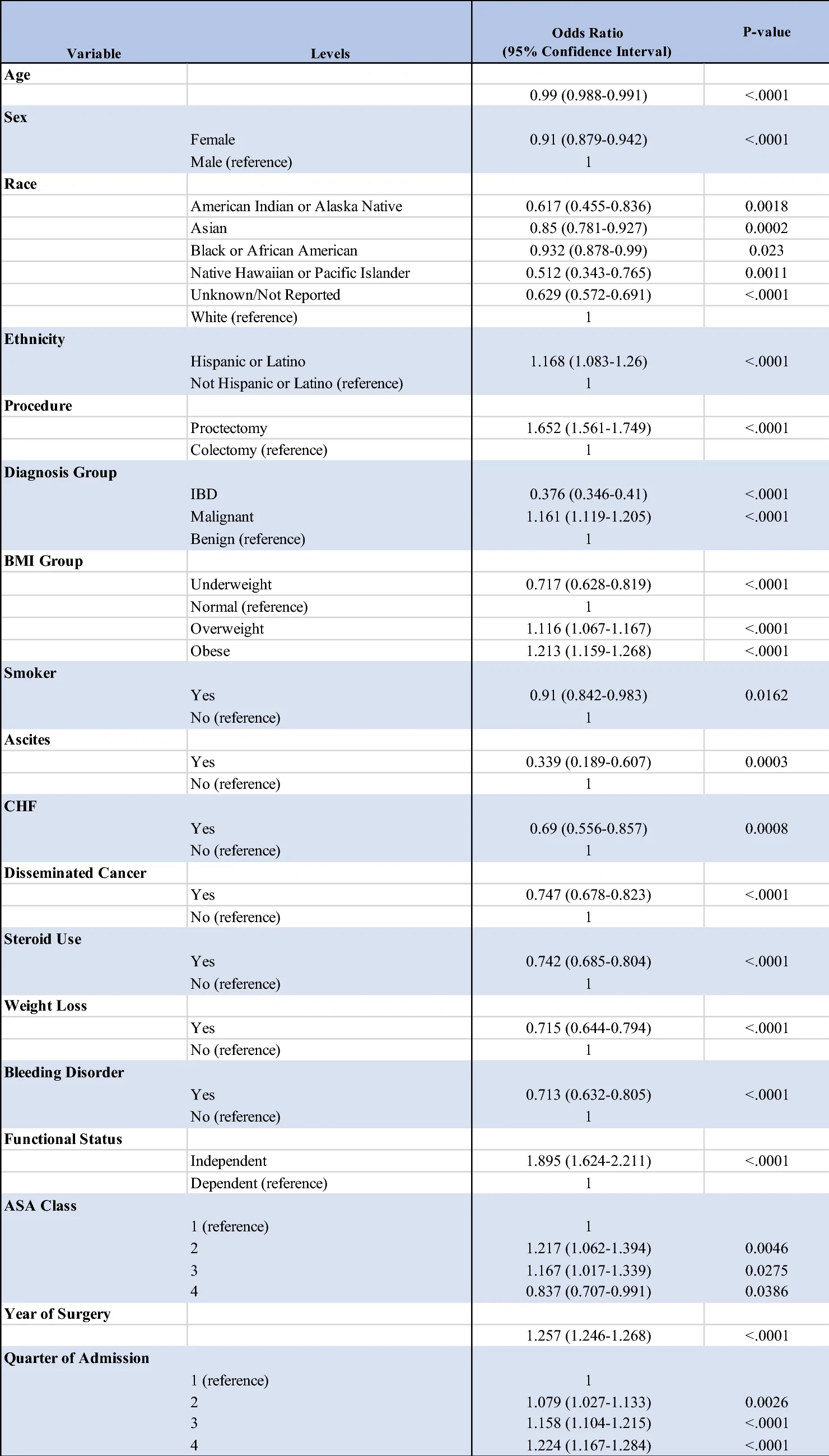
NSQIP multivariate analysis: odds of having undergone robotic vs non-robotic (open or laparoscopic) surgery in Whites versus individual non-White races

**Table 8:**
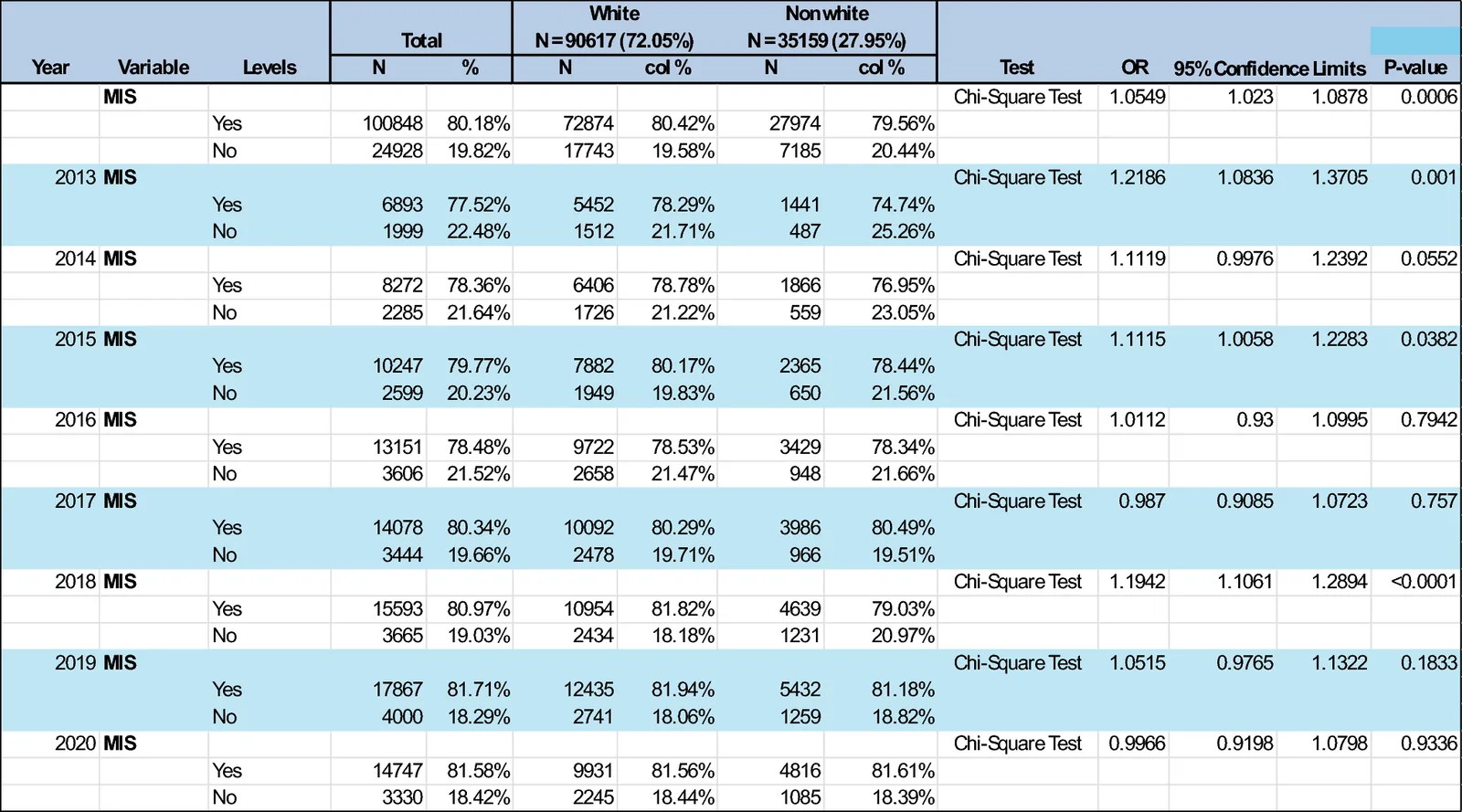
NSQIP multivariate analysis: odds of having undergone MIS surgery in Whites versus individual non-White races

**Table 9:**
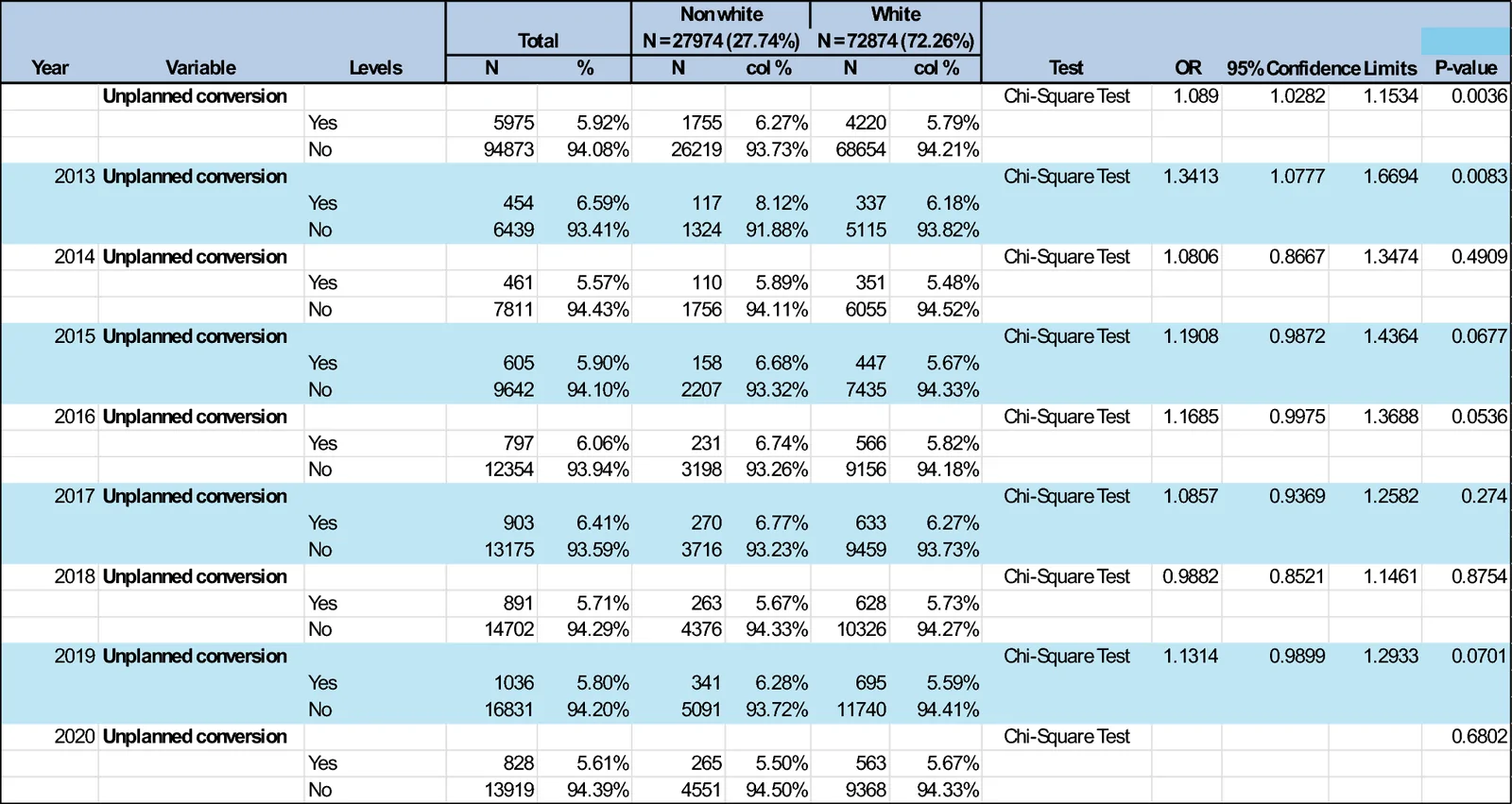
NSQIP multivariate analysis: odds of having undergone unplanned conversion to open in individual non-White races versus Whites

## Postoperative outcomes in robotic versus non-robotic surgery

Multivariate analysis of reported 30-day postoperative complications demonstrated that robotic surgery benefited patients when compared to surgeries performed non-robotically (Table 10). After RAS, patients were less likely to have a postoperative complication (OR 0.86, CI 0.82–0.9) or a serious adverse event (OR 0.9, CI 0.84–0.97) and had lower odds of 30-day mortality (OR 0.72, CI 0.57–0.91). Individual complications that were less likely after robotic surgery included superficial incisional infection, wound disruption, pneumonia, unplanned intubation, DVT requiring therapy, transfusion requirement intra- or postoperatively, ileus and sepsis. In contrast, need for reoperation was more likely to occur after robot-assisted surgery compared to non-robotic approaches. Furthermore, the differences in outcomes still exist when separating robotic versus laparoscopic and robotic versus open (Tables 11 and 12). A list of the covariates can be found in Appendix 2.

**Table 10:**
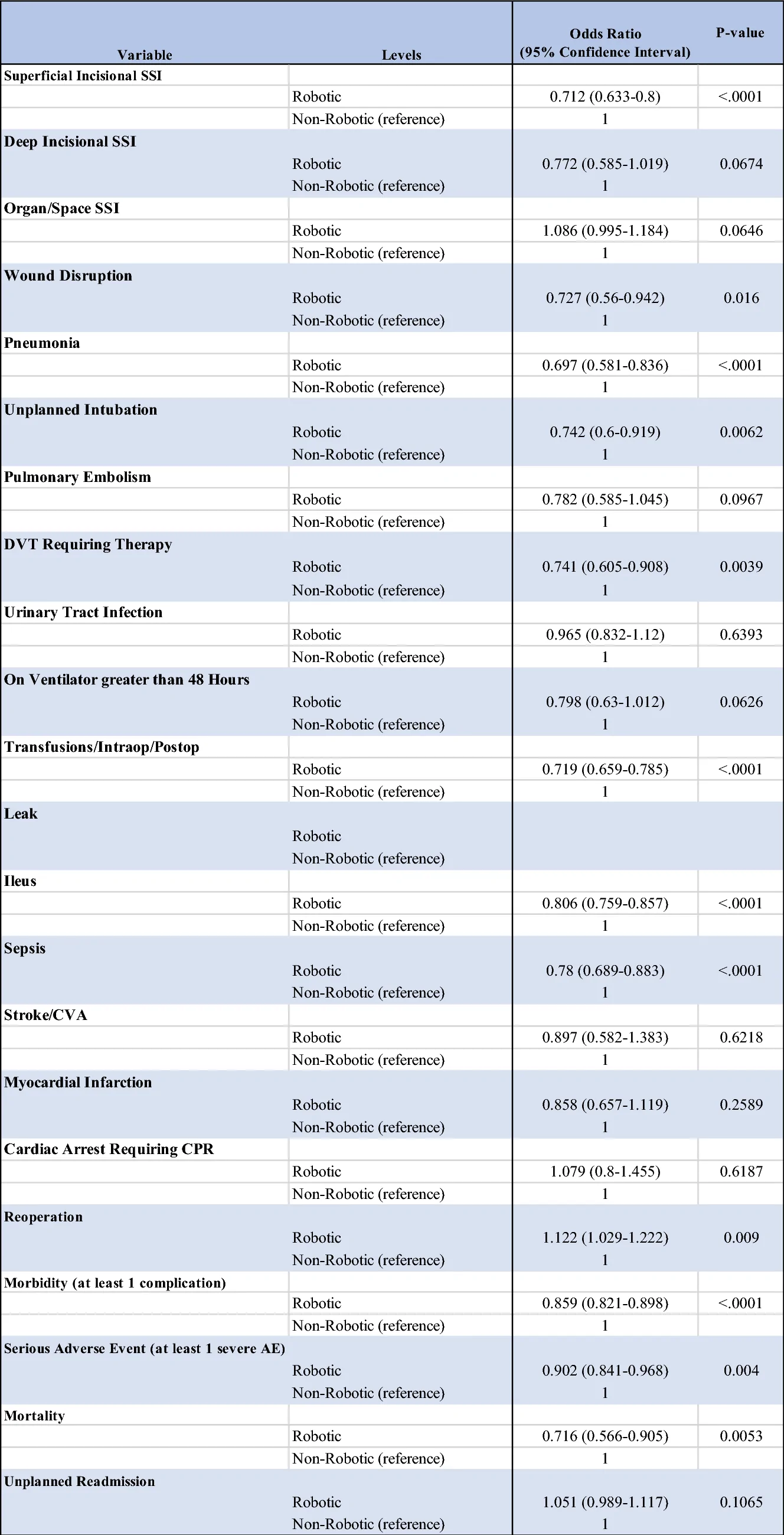
NSQIP multivariate analysis: odds of postoperative complications in robotic vs non-robotic cases

**Table 11:**
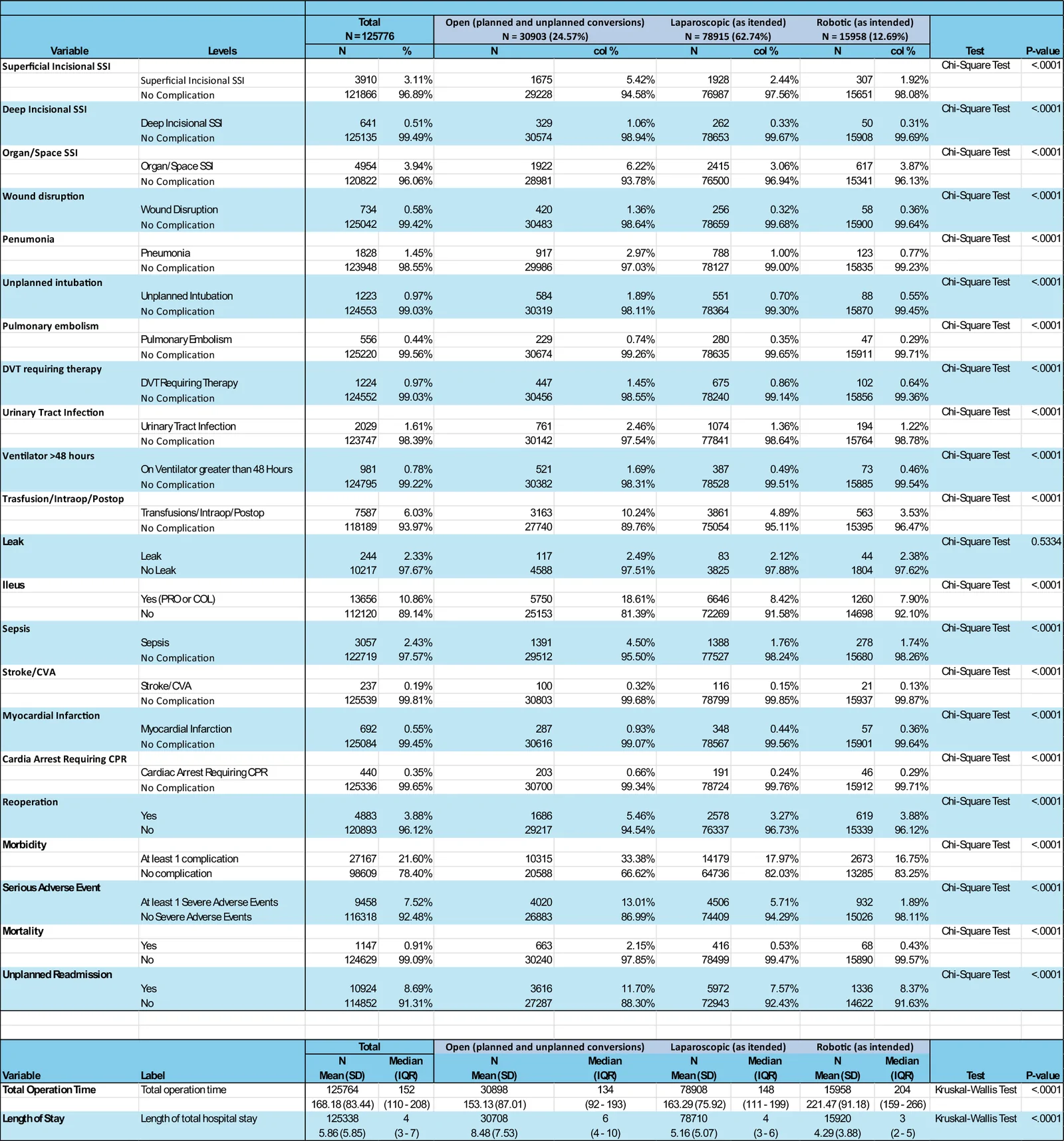
Odds of postoperative complications in open, laparoscopic, and robotic surgery

**Table 12:**
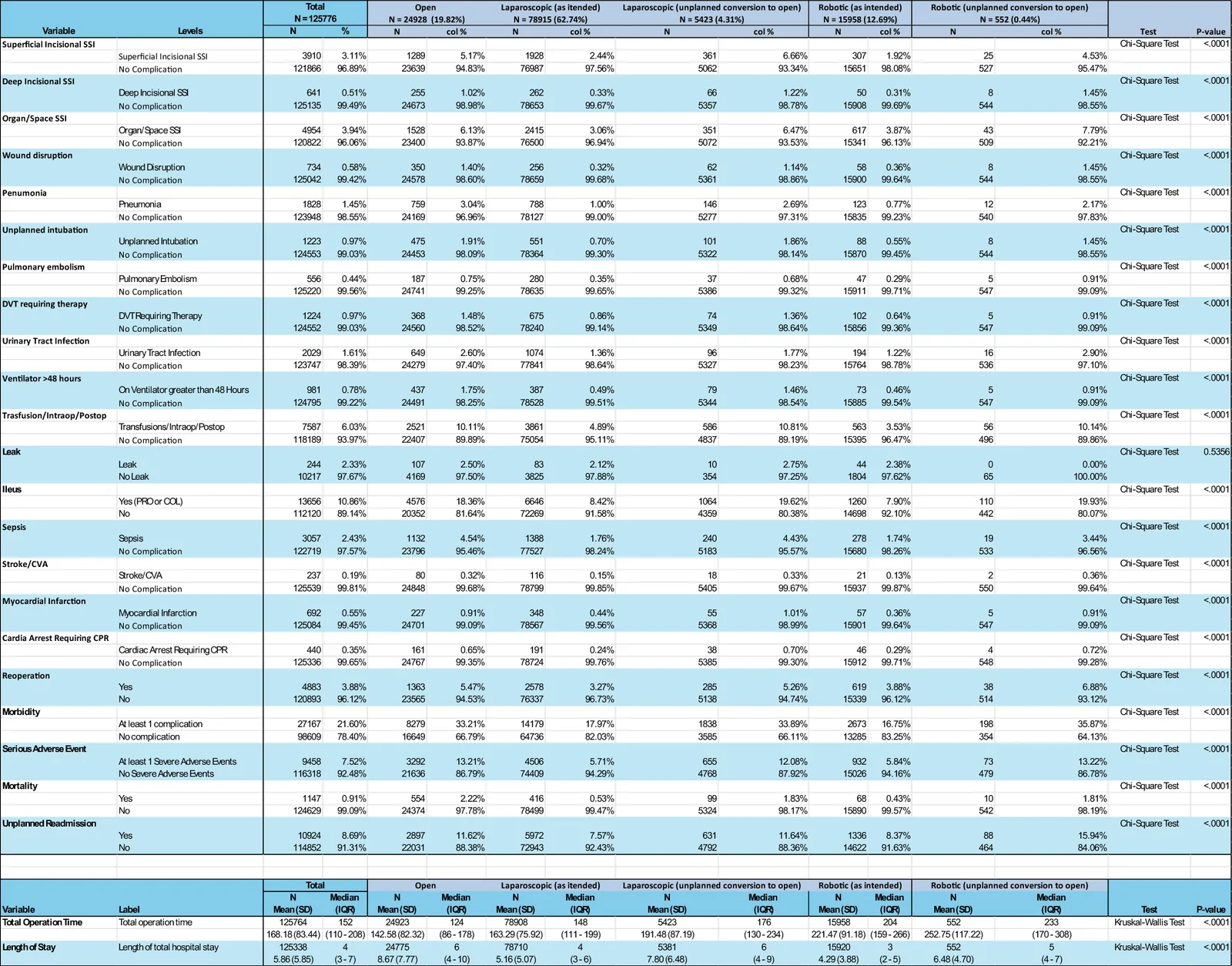
Odds of postoperative complications in laparoscopic and robotic with conversion to open

## Discussion

Robot-assisted surgery has been increasing in overall use and in its application to colorectal surgery over the last two decades, with studies showing up to a 158% increase in RAS from 2011 to 2015 [5, 13]. The robotic platform appears to provide benefit to both the surgeon and the patient. Improved ergonomics and the numerous technical aspects of the robot make complex dissections more approachable, which is especially important for low pelvic dissections in rectal surgery. The literature has shown that RAS yields largely equivalent patient outcomes compared to laparoscopy, although long-term studies are lacking. Some short-term studies have demonstrated superiority of RAS, citing shorter hospital LOS, lower conversion rate, lower rate of circumferential margin involvement for rectal cancers and lower overall complications [12]. However, adoption of robotic platforms has not come without challenges, which include high overhead cost and mastery of a new skillset, amongst others. These hurtles contribute to an unbalanced distribution of robotics across the country, favoring metropolitan hospitals and academic programs. Unfortunately, we are now seeing racial and socioeconomic disparities in RAS as well.

In this study, we queried a national database for colorectal resections from 2013–2020 with the intention of evaluating the progression of these disparities over time. Over the entire study period, we found that females and non-White patients were significantly less likely to undergo RAS. In sub-analysis of each individual race compared to White patients, all were associated with lower likelihood of robotic surgery. When we analyzed the data year by year, we found that the gap in RAS access between White and non-White patients had significantly widened from 2014–2020. In 2014, a White patient’s odds of undergoing RAS were 1.51, which increased to 1.96 in 2020. It is very clear than on an annual basis, the percentage of operations being performed robotically is growing much faster in White versus the non-White populations. We obtained similar results after exclusion of open cases: non-White patients were increasingly less likely to undergo RAS even when compared to laparoscopy alone as time went on. As shown in Appendices 3 and 4, evaluation of individual races at the annual level yielded very few results that were statistically significant. The only subgroup that demonstrated consistent significance was the race Unknown/Not Reported group, from which drawing any meaningful conclusions is challenging.

Our findings confirm what has been consistently demonstrated in the literature. Even before the introduction of RAS, Black patients have a history of reduced access to MIS in the treatment of benign and malignant colorectal diseases [19, 20]. This trend was first observed in laparoscopy and has unfortunately become the case with robotics, as demonstrated in multiple studies [22, 24]. Although RAS is still a relatively new innovation, it offers benefits that seem to be at least equivalent to those afforded by laparoscopy. In a 2017 commentary by Berkley and Gabriel, the authors made the very salient point that disparities in access to RAS may result in disparities in access to the associated benefits [23]. Our multivariate analysis suggests that RAS benefits patients in both morbidity and mortality: the odds of developing at least one postoperative complication or serious adverse event and of 30-day mortality were all decreased (OR 0.86, 0.9, and 0.72, respectively) in the robotic group. Many postoperative complications were significantly less likely after RAS (Table 8). The unequitable use of RAS in non-White patient populations may at least partially contribute to those patients experiencing disproportionately higher rates of complications and mortality.

Our study has both strengths and limitations. Gathering data from a validated, national database over a several-year period resulted in a large study population, allowing numerous iterations of analysis to be performed with sufficient statistical power. We suspect power became an issue when we attempted annual sub-analysis of individual races, as most of our analyses yielded p-values > 0.05. Perhaps with larger populations these findings may have demonstrated significance. We recognize the limitations inherent to retrospective analysis, most notably the lack the randomization afforded by a prospective study design. For this reason, multivariate analysis was crucial, and we believe the listed covariates encompassed many of the most common comorbidities encountered in surgical patients. The use of large national databases is limited by the coding performed by administrators and errors could occur.

Finally, our study is limited by the data points included in the NSQIP database. Importantly, we were unable to control for cancer stage given staging information in NSQIP is not of high quality or is often missing. Non-White patients often present with more advanced cancer; however, it is unclear whether these patients were not offered MIS options based on stage. In this study, non-White patients were more likely to undergo unplanned conversion to open surgery. This could imply that they have more advanced disease leading to more technically difficult operations requiring conversion to an open approach. Future studies should investigate how cancer stage affects utilization of RAS in different patient populations. In addition, our study was focused on patient demographics, basic surgical details and short-term outcomes, for which the NSQIP database is curated but does not have the capability to assess mediating factors that racial groups may be affected by (i.e. social determinants of health, environmental factors, access to health care).

## Conclusions

With a growing body of research addressing disparities in access to healthcare and health outcomes, the hope is that awareness of these issues will lead to tangible change. Some studies, however, have demonstrated that many institutions still do not have efforts in place to address disparities in delivery of care [25]. While awareness of a problem is the first step in solving it, it is not tantamount to action. The literature is populated with reports of racial disparities in surgical care, yet our findings suggest that the gap between White and non-White patients’ access to our most advanced surgical therapies is widening. With disparities already acknowledged in the literature, the next step should be investigating deliberate interventions to narrow this gap. Equitable access to robotic surgery is not only imperative, it can be a focused, feasible first step in improving disparate outcomes in colorectal surgery.

## Appendix 1 ICD-9 and -10 codes used for NSQIP search

**Table:**

	ICD-9 and -10 Codes
Benign diagnosis	**008.49, 038.9** 209.43, 209.5, 209.50, 209.51, 209.52, 209.53, 209.54, 209.55, 209.56, 209.57, 209.60, 211.3, 211.4, 214.3, 230.3, 230.4, **235.2, 239.0,** 540, 540.0, 540.1, 540.9, 541, 542, 543, 543.0, 543.9, 557, 557.0, 557.1, 557.9, 558, 558.1, 558.2, 558.42, 558.9, 560, 560.0, 560.1, 560.2, 560.3, 560.30, 560.31, 560.32, 560.39, 560.8, 560.81, 560.89, 560.9, 56,081, 562, 562.0, 562.00, 562.01, 562.02, 562.03, 562.1, 562.10, 562.11, 562.12, 562.13, 564, 564.0, 564.00, 564.01, 564.02, 564.09, 564.1, 564.4, 564.7, 564.81, 564.89, 564.9, 565.1, 566, 569.0, 569.1, 569.2, 569.3, 569.41, 569.42, 569.49, 569.5, 569.6, 569.60, 569.6, 569.62, 569.69, 569.71, 569.82, 569.83, 569.84, 569.85, 569.86, 569.89, 569.9, 578.9, 596.1, **619.1,** V44.2, V44.3, V55.2, V55.3, A04.7, **A41.9,** D01.0, D01.1, D01.2, D12.0, D12.1, D12.2, D12.3, D12.4, D12.5, D12.6, D12.7, D12.8, D17.5, D17.79, D17.79, D17.9, D18.03, D37.4, D37.5, K56.2, K56.6, K56.60, K56.69, K57 (all), K62.0, K62.1, K62.3, K62.4, K63.2, K63.5, K63.89, 64.8, N32.1, N82.3, N82.4, Z43.2, Z43.3
Malignant diagnosis	152.2, 152.8, 152.9, 153, 153.0, 153.1, 153.2, 153.3, 153.4, 153.5, 153.6, 153.7, 153.8, 153.9, 154, 154.0, 154.1, **154.8, 209.11, 209.03,** 197.5, 197.6, C17.2, C18, C18.0, C18.1, C18.2, C18.3, C18.4, C18.5, C18.6, C18.7, C18.8, C18.9, C19, C20, C21.8, C49.A4, C49.A5, C78.5, C78.6, C7A.012, C7A.020, C7A.021, C7A.022, C7A.023, C7A.024, C7A.025, C7A.026, C7A.029
Inflammatory bowel disease	555.0, 555.1, 555.2, 555.9, 556, 556.0, 556.1, 556.2, 556.3, 556.4, 556.5, 556.6, 556.8, 556.9, K50 (all), K51 (all)
	**CPT Codes**
Colectomy and proctectomy	44,140, 44,141, 44,143, 44,144, 44,145, 44,146, 44,147, 44,150, 44,151, 44,160, 44,204, 44,205, 44,206,44,207,44,208,44,210, 44,155, 44,156, 44,157, 44,158, 44,211, 44,212, 45,110, 45,111, 45,112, 45,113, 45,114, 45,116, 45,119, 45,120, 45,121, 45,123, 45,126, 45,130, 45,135, 45,550, 57,307, 45,395, 45,397, 45,402

## Appendix 2 List of covariates for all multivariate analyses

**Table:**

**Covariates**
Age (as continuous variable, 90 +−> 90)
Sex
Race (White vs. non-White or 6 race categories)
Ethnicity
BMI
Smoker
Diabetes
Hypertension
COPD
Ascites
Congestive heart failure
Dialysis
Disseminated cancer
Steroid use
Weight loss
Bleeding disorder
Functional status
ASA
Year of surgery (as continuous variable)
Quarter of admission
Diagnosis group
Procedure (proctectomy vs. colectomy)

## Abbreviations

ASA: American Society of Anesthesiologists
CI: Confidence interval
CPT: Current Procedural Terminology
IBD: Inflammatory bowel disease
ICD: International Classification of Diseases
LOS: Length of stay
MIS: Minimally invasive surgery
NSQIP: National Surgical Quality Improvement Program
OR: Odds ratio
RAS: Robot-assisted surgery
SSI: Surgical site infection
